# Altered striatal functional connectivity and structural dysconnectivity in individuals with bipolar disorder: A resting state magnetic resonance imaging study

**DOI:** 10.3389/fpsyt.2022.1054380

**Published:** 2022-11-09

**Authors:** Charles Okanda Nyatega, Li Qiang, Mohammed Jajere Adamu, Halima Bello Kawuwa

**Affiliations:** ^1^School of Electrical and Information Engineering, Tianjin University, Tianjin, China; ^2^Department of Electronics and Telecommunication Engineering, Mbeya University of Science and Technology, Mbeya, Tanzania; ^3^School of Microelectronics, Tianjin University, Tianjin, China; ^4^Department of Biomedical Engineering, Tianjin University, Tianjin, China

**Keywords:** bipolar disorder, gray matter, fMRI, functional connectivity, striatum, sliding windows

## Abstract

**Objective:**

Bipolar disorder (BD) is a mood swing illness characterized by episodes ranging from depressive lows to manic highs. Although the specific origin of BD is unknown, genetics, environment, and changes in brain structure and chemistry may all have a role. Through magnetic resonance imaging (MRI) evaluations, this study looked into functional abnormalities involving the striatum between BD group and healthy controls (HC), compared the whole-brain gray matter (GM) morphological patterns between the groups and see whether functional connectivity has its underlying structural basis.

**Materials and methods:**

We applied sliding windows to functional magnetic resonance imaging (fMRI) data from 49 BD patients and 44 HCs to generate temporal correlations maps to determine strength and variability of the striatum-to-whole-brain-network functional connectivity (FC) in each window whilst also employing voxel-based morphometry (VBM) to high-resolution structural MRI data to uncover structural differences between the groups.

**Results:**

Our analyses revealed increased striatal connectivity in three consecutive windows 69, 70, and 71 (180, 182, and 184 s) in individuals with BD (*p* < 0.05; Bonferroni corrected) in fMRI images. Moreover, the VBM findings of structural images showed gray matter (GM) deficits in the left precentral gyrus and middle frontal gyrus of the BD patients (*p* = 0.001, uncorrected) when compared to HCs. Variability of striatal connectivity did not reveal significant differences between the groups.

**Conclusion:**

These findings revealed that BD was associated with a weakening of the precentral gyrus and middle frontal gyrus, also implying that bipolar illness may be linked to striatal functional brain alterations.

## Introduction

Bipolar disorder (BD) is a chronic, severe, and fluctuating mental disorder, and the worldwide prevalence of the BD spectrum is 1–4% (1). It is a mental illness that causes dramatic shifts in a person’s mood and energy (2). The most common subtypes of bipolar disorder are I and II, characterized by mood swings between depression and mania (bipolar I) or hypomania (bipolar II), followed by a period of emotional remission known as euthymia (3).

The study conducted by Harvard Medical School (2007) based on diagnostic interview data from National Comorbidity Survey Replication (NCS-R) shows that an estimated 2.8% of US adults had BD in 2006 (2.9% males and 2.8% females), and nearly 83% of cases were classified as severe (4). Although researchers agree that BD is multifactorial with genetic and environmental risk factors, the neuropathological mechanisms remain unclear (5). BD is characterized by difficulty in regulating the pursuit of goals (6), and onset of manic and depressive episodes linked to goal achieving failure (7, 8). In addition to an increased risk of suicide, BD is also associated with considerable medical comorbidities, including cardio- and cerebrovascular disease, and metabolic and endocrine disorders, which, when combined with neuropsychiatric morbidity and suicidality, it reduces life expectancy by an average of 11 years in females and 10 years in males afflicted with bipolarity (9–11).

**FIGURE 1:**
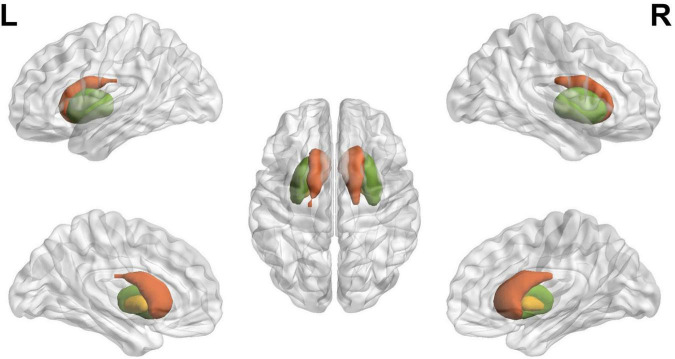
The schematic diagram of striatum, where red, green, and yellow indicate caudate, putamen, and pallidum, respectively, defined using the BrainNet viewer.

A recent study suggests that the pattern of functional activation in specific brain regions may serve as a potential biomarker that distinguishes BD from other conditions with comparable clinical symptomatology, such as major depressive disorder (12) and schizophrenia spectrum disorders (13). This demonstrates the potential value of investigating the functional connectivity of a specific network of brain regions as an effective way to comprehend bipolar disease (14). The current study was designed to understand the role of the Striatum (caudate, putamen, and pallidum, as seen in Figure 1) during the course of BD. This region is part of the brain that coordinates many primary aspects of behavior such as motor and action planning, motivation, and rewarding perception (15). The caudate nucleus regulates cognitive control processes by interacting with dorsolateral prefrontal cortex (16). The regulation of motor function is achieved by Putamen through interaction with motor-cortices (17). The three basic operations that are commonly associated with the striatum are action preparation, response or motor-set selection and learning (18).

It can be observed that BD altered behaviors are fundamentally linked with striatum processes. Thus, understanding dynamic changes in striatal functional connectivity might provide some insights to the underlying mechanisms of BD. In support of our hypothesis, Haznedar et al. (19) showed that psychotic and mood symptoms in BD were related to alterations in limbic cortex-basal ganglia-thalamo-cortical circuit. Cognitive deficits were also related to altered striato-prefrontal circuits of BD (20). One study detected limbic striatal volume atrophy and thicker putamen in patients with bipolar I (21) and bigger left putamen volume in bipolar II patients compared to bipolar I patients in another study (22). BD subjects also demonstrated considerably increased left striatal activity in response to mild happy faces when compared to HC subjects (23) the reason being that striatal regions are thought to be involved in the perception of potentially rewarding stimuli such as food (24). Recent study by Karcher et al. of striatum seed-based analysis revealed impaired ventral rostral putamen connectivity with the salience network portion of the medial prefrontal cortex in both schizophrenia and psychotic BD (25). Clinical aspects such as anhedonia have been linked to the reduced responsivity of the ventral striatum to reward stimuli in BD (26). Together these findings demonstrate the role of striatum in BD.

Although methodologically contrary to this study, previous studies of functional connectivity (27) have detected altered functional organizations in BD (28) such as reward circuit (29). These static FC alterations during mood episodes were found in the default mode network (DMN), limbic, subcortical (striatal), and cerebellum networks (5, 30–36). These studies regarded the FC properties of the entire rs-fMRI scan as stationary not dynamic. The growing evidence suggest that the human brain functional processes are inherently dynamic, and thus capturing temporal variations of these processes might be essential for understanding neuropathology. Shorter time windows can be sensitive enough to capture these temporal fluctuations of functional connectivity that may be related to pathology of BD (37–40). Recently, the new evidence has shown abnormal dynamic functional connectivity in patients with BD and Schizophrenia (28, 41–44). However, we expect that some connectivity measurements might be better recorded in a static model, while others would be better captured in a dynamic one (45). Static functional connectivity gives information about overall mean connectivity and may be preferable to a dynamic approach for connectivity that persists during the experiment (43). Dynamic functional connectivity, on the other hand, will be better at capturing information on changes in local connectivity at different time windows (43, 46). As a result, we believe that both static and dynamic functional connectivity approaches capture complimentary aspects of connectivity, and that combining their features will increase classification performance beyond what each type of feature can do on its own (43, 47, 48). In this work, we present a static connectivity within time window.

However, the changes in striatal functional connectivity that are hypothetically linked with BD are yet to be explored, providing the necessity of the present study. In an attempt to investigate these striatal alterations in BD, we applied a series of windows on resting-state fMRI data to construct dynamic correlation maps, and assessed the strength and variability of these dynamic connectivity maps within each temporal window. Finally, we evaluated group differences between BD (49 participants) and Healthy Controls (44 participants). In order to understand whether there are abnormalities in both the functional connectivity and gray matter connections between brain regions, in this study we performed both functional connectivity and structural methods, raising the possibility that structural abnormalities may be responsible for functional connectivity abnormalities in the disorders. We used DARTEL VBM to detect structural gray matter (GM) alterations in patients with BD in which is images from multiple participants are normalized (contrast stretched) and registered to produce a brain atlas or template that represents a particular collection of subjects (49). This was done by employing the Computational Anatomy Toolbox (CAT12) in T1 images, which is an add-on to the SPM12 (Statistical Parametric Mapping) software package.

## Materials and methods

### Resting state functional magnetic resonance imaging data

In this study, we used a dataset of rs-fMRI images obtained from patients with BD and HC control. These data were obtained from the OpenfMRI database with accession number ds000030^1^ (50). We used all available 49 subjects in the BD dataset and 44 subjects in the HC group. The inclusion criteria for subjects were of 52 men and 41 women with ages between 21 and 50 years age. Each subject completed at least 8 years of formal education and have either English or Spanish as primary language. Subjects were recruited by community advertisement and through outreach to local clinics and online portals. Furthermore, the following exclusion criteria were used: history of significant medical illness, contraindications for MRI (including pregnancy), any mood-altering medication on scan day (based on self-report), vision that was insufficient to see task stimuli, and left-handedness. Participant’s demographics, primary diagnosis information for BD patients and medications are presented in Tables 1, 2.

**TABLE 1:** Participant demographics and primary diagnosis information for bipolar disorder (BD) patients.

	BD (*n* = 49)	HC (*n* = 44)	*P*-value
Age (years)	35.29 ± 9.03	33.27 ± 8.90	0.281^1^
Sex (males/females)	28/21	24/20	0.801^2^
Males	35.89 ± 9.22	33.25 ± 9.42	0.313^1^
Females	34.48 ± 8.93	33.30 ± 8.47	0.667^1^

**Diagnosis (DSM code)**			**Number**

BP I, most recent episode hypomanic (296.40)			4
BP I, most recent episode manic, mild (296.41)			2
BP I, most recent episode manic, moderate (296.42)			1
BP I, most recent episode manic, in partial remission (296.45)			3
BP I, most recent episode manic, in full remission (296.46)			6
BP I, most recent episode depressed, mild (296.51)			2
BP I, most recent episode depressed, moderate (296.52)			4
BP I, most recent episode depressed, severe without psychotic features (296.53)			5
BP I, most recent episode depressed, in partial remission (296.55)			8
BP I, most recent episode depressed, in full remission (296.56)			5
BP I, most recent episode mixed, moderate (296.62)			1
BP I, most recent episode mixed, severe with psychotic features (296.64)			3
BP I, most recent episode unspecified (296.70)			5

^1^Two-sample *t*-test.

^2^Chi-square test.

DSM, diagnostic and statistical manual of mental disorders, Data are shown in mean ± SD.

**TABLE 2:** Bipolar disorder (BD) patients by medication table (110).

Anticonvulsant-mood stabilizer	Psychostimulant	Antidepressant	Antipsychotic	Sedative-Hypnotic	Analgesic	Hormone	Anxiolytic	Antiparkinsonia	Anticholinergic	Other

30	4	15	22	3	2	2	6	1	0	4

Neuroimaging data were acquired on a 3T Siemens Trio scanner. Functional MRI data were collected with a T2*-weighted echoplanar imaging (EPI) sequence with parameters: slice thickness = 4 mm, 34 slices, TR = 2 s, TE = 30 ms, flip angle = 90°, matrix = 64 × 64, FOV = 192 mm. A T1-weighted high-resolution anatomical scan (MPRAGE) were collected with the following parameter: slice thickness = 1 mm, 176 slices, TR = 1.9 s, TE = 2.26 ms, matrix = 256 × 256, FOV = 250 mm. The resting fMRI scan lasted 304 s.

### Data preprocessing

Data preprocessing was achieved using Data Processing and Analysis for Brain Imaging (DPABI V5.0,^2^) (51), an open-source package based on Statistical Parametric Mapping (SPM12)^3^ and MATLAB (MATLAB and Statistics Toolbox Release 2018b, The Mathworks, Inc., Natick, MA, United States).

The steps for data preprocessing were as follows: (i) removing the first five volumes to allow magnetization stabilization (ii) correcting slice-timing and realigning images; (iii) manually reorienting structural and functional images; (iv) co-registering structural images into functional images and segmenting to gray matter, white matter, and cerebrospinal fluid; (v) regressing nuisance covariates (including Friston 24 head motion parameters (52) and white matter and cerebrospinal fluid signals); (vi) Normalizing functional images to Montreal Neurological Institute standard space by Diffeomorphic Anatomical Registration Through Exponentiated Lie algebra Method (DARTEL) (53) and reslicing to 3.0 mm^3^ × 3.0 mm^3^ × 3.0 mm^3^; (vii) performing spatial smoothing (Gaussian kernel of 6 mm FWHM); (viii) band-pass filtering (0.01–0.08 Hz) to reduce the effects of low-frequency signals and high-frequency aliasing after data normalization and (ix) scrubbing image volumes with FD (Jenkinson) > 0.2 mm to reduce the effect of head motion using cubic spline interpolation (54, 55). Subjects were not excluded as they did not exceed the head transition < 3 mm, rotation < 3°(56).

### Regions of interest definition and network

We used the regions from the automated anatomical labeling template (AAL) (57), to calculate the functional connectivity (FC) based on region of interest (ROI) analysis, dividing the 90 ROIs of AAL (without cerebellum) into six main regions (including prefrontal regions, other regions of frontal lobe, parietal regions, occipital regions, temporal regions, and subcortical regions according to prior studies (58). Finally, we extracted mean time courses from all 90 ROI’s to calculate functional connectivity.

### Resting state functional connectivity analysis

**FIGURE 2:**
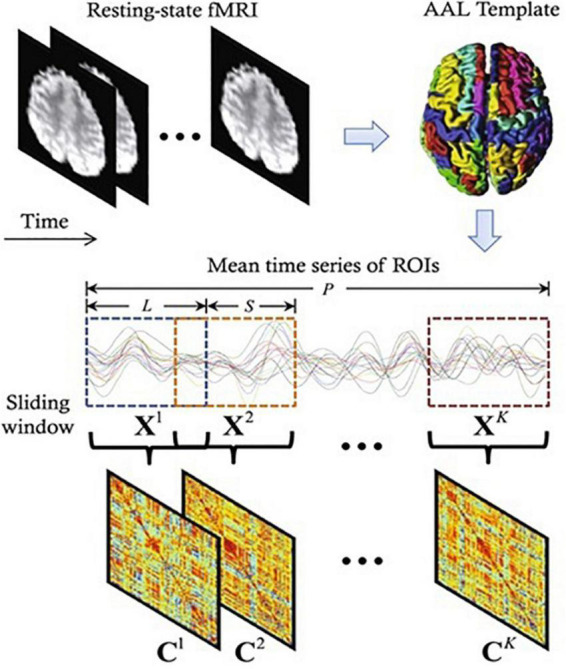
A diagram depicting the construction of functional connectivity (FC) networks using sliding window-based Pearson’s correlation on rs-fMRI data (65). Where *P, L, S, C*, and *X* represents time points, window length, step size, correlation strength, and number of windows, respectively.

For the construction of static rsFC analysis network of the brain, we computed Pearson’s correlation coefficients between each pair of the averaged time course in 90 ROIs, we used Fisher *z*-transformation to convert *r* into *z* values to improve the normality of correlation distribution which is over a full range of 147-image volumes, to stabilize variance prior to further analysis. For the shorter time analysis, the sliding window approach was used (59). To date, the most widely used method for evaluating rsFC in smaller time-series is the sliding window, in which, the fMRI data is segmented into overlapping windows and the functional interconnection between different brain regions within each window is evaluated (37, 59–61). Comparisons of window sizes revealed that 44 s offers a solid balance between the ability to handle dynamics and the efficiency of covariance matrix evaluation, which is consistent with demonstrations that cognitive states can be correctly identified using covariance matrices estimated on as little as 30–60 s of data (62) and that structural brain network evaluations begin to improve at window lengths of around 30 s (63). In our study we employed a sliding temporal window of 22 TRs (44 s) to 147 data length (294 s), rectangular sliding windows unconvolved with Gaussian kernel was then used to capture more sharp transitions that could be undetected in tapered windows (64). By sliding the window by the 2-s step size 1TR, 126 temporal windows (147 – 22 + 1) were generated. Lastly, we obtained 126 Fisher’s *z*-transformed Pearson’s correlation maps (90 × 90 matrix size, for each window for each subject which were the rsFC maps, as seen in Figure 2 (65). Having obtained these maps during parameter computations, we generated Matlab codes to observe striatal functional connectivity in each of these 126 windows by computing strength and variability within each temporal windowed connectivity map to express its characteristics.

### Voxel-based morphometry

**FIGURE 3:**
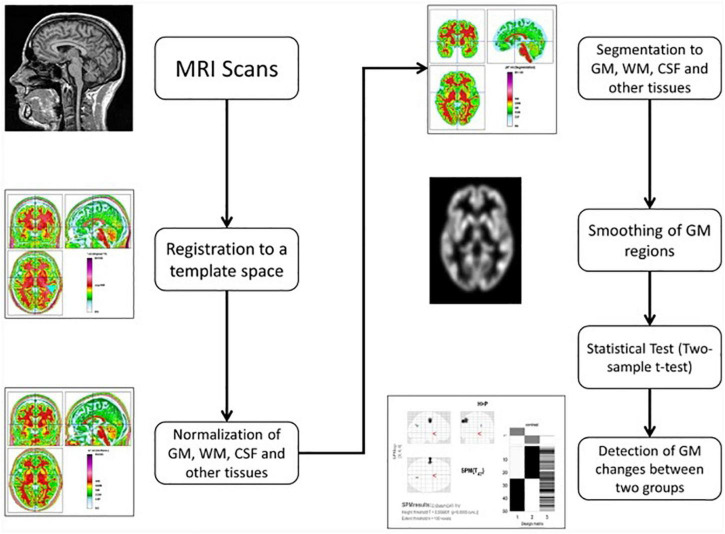
The processing framework of voxel-based morphometry (VBM) analysis using the CAT12 toolbox of SPM12 software as depicted by Seyedi et al. (67).

We employed CAT12 toolbox implemented in SPM12 software and run it in MATLAB for VBM analysis. All 3D T1-weighted Neuroimaging Informatics Technology Initiative (NIFTI) MR images were spatially normalized and segmented into GM, WM, and CSF tissue classes according to the DARTEL approach with default settings in 1.5 mm cubic resolution and MNI space. To preserve GM volumes of native space, the normalized maps were modulated with the resulting Jacobian determinant maps and smoothed using an 8-mm FWHM Gaussian kernel. In the CAT12 toolbox, the procedures of segmentation, normalization, and modulation were all done automatically. Total intracranial volume (TIV) and the native space volumes of GM, WM, and CSF maps were estimated with TIV as a covariate of no interest. The two-tailed *t* test was then produced using family-wise error (FWE) correction and a *p* < 0.05 threshold, as well as uncorrected *p* = 0.001 thresholds. The 100 voxel extent threshold was chosen and finally we used *xjview* (66) toolbox for MATLAB to record voxel brain area (represented with pseudo color),with significant differences, activation volume (cluster), activation intensity (statistically analyzed with *t*-test and expressed as *T* value; *T* value is proportional to the intensity). Figure 3 (49, 67) depicts the VBM analysis processing framework.

### Functional magnetic resonance imaging statistical analysis

For the fMRI statistical analyses, we performed multiple comparison correction using Gretna toolbox^4^ with a threshold of uncorrected *p* < 0.05. A 5,000-times randomized permutation test was used. A permutation test is a kind of statistical significance test in which all potential values of the test statistic under rearrangements of the labels on the observed data points are calculated to obtain the distribution of the test statistic under the null hypothesis (68). The regions that made it through multiple comparison correction were chosen as region of interest (ROIs) for post hoc analysis. On these ROIs, a two-tailed, two-sample *t*-test was used to detect the differences between the groups (BD vs HC). Statistical significance was defined as a *p* < 0.025 (0.05/2) (Bonferroni corrected) value.

## Results

### Participants’ demographic and neuropsychological evaluation

Tables 1, 2 show subjects’ clinical information and BD group patient’s medication in which no significant difference was seen between age and sex of the two groups (*p* > 0.05). Gender was analyzed by chi-square test; other variables were analyzed by independent samples *t*-test.

### Resting state functional connectivity

**FIGURE 4:**
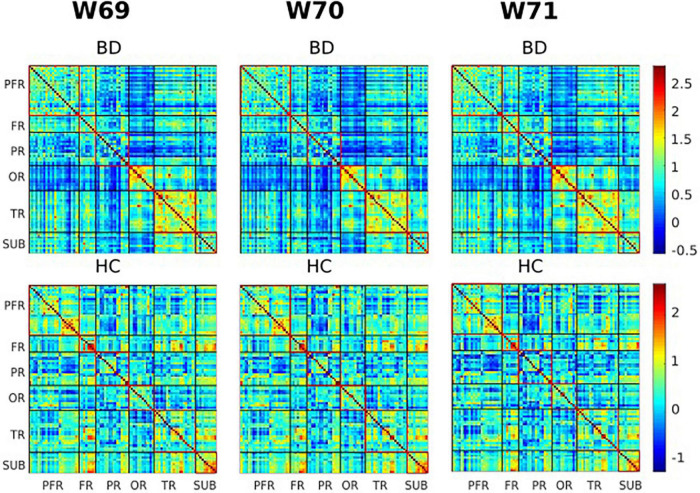
Visualization of functional connectivity maps of one subject from the bipolar disorder (BD) and healthy controls (HC) group, respectively, at windows 69, 70, and 71 (180, 182, and 184 s). Values are plotted as –log10 (*p*-value) × sign (*t*-statistic). The lines partition the rsFC maps into six subcategories (i.e., PFR, prefrontal; FR, other frontal; PR, parietal; OR, occipital; TR, temporal; SUB, subcortical regions). The color bars represent correlation (*z*-scores).

**FIGURE 5:**
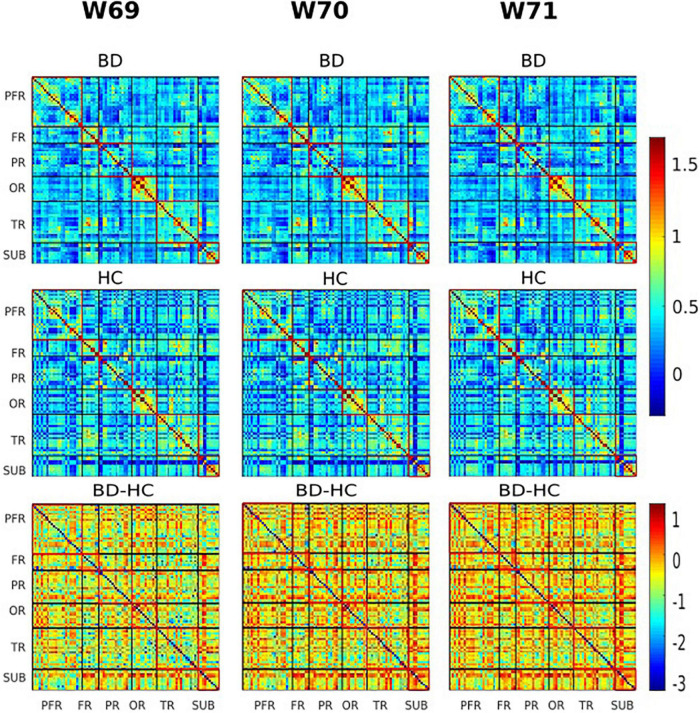
The group variability maps at windows 69, 70, and 71 (180, 182, and 184 s). Where BD-HC is the group difference that survived the thresholding, values are plotted as –log10 (*p*-value) × sign (*t*-statistic). The lines partition the rsFC maps into six subcategories (i.e., PFR, prefrontal; FR, other frontal; PR, parietal; OR, occipital; TR, temporal; SUB, subcortical regions). Color bars represent correlation (*z*-scores).

Of the two metrics to assess rsFC, striatal functional connectivity demonstrated a significant difference (*p* < 0.05) between BD patients and HC group in three consecutive windows w69, w70, and w71 (180, 182, and 184 s) during scanning time (Figures 4, 5). In particular, when compared to healthy controls, BD patients presented increased striatal functional connectivity in these windows with significant between-group striatal-rsFC difference (*p* = 0.023, 0.019, and 0.022). In contrast to strength, the variability of striatal connectivity did not reveal any significant difference (*p* > 0.05) between the groups.

### The voxel-based morphometry analysis

**FIGURE 6:**
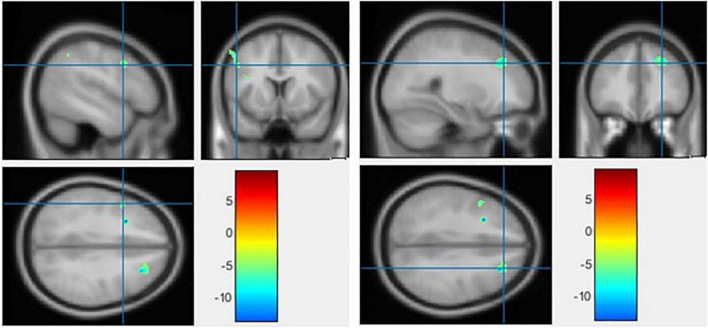
The significant gray matter (GM) alterations by voxel-based morphometry (VBM) analyses with the covariate of no interest (TIV) in the left precentral gyrus **(left)** and middle frontal gyrus **(right)**, respectively, when BD < HC with *p* < 0.001 (uncorrected) and extent threshold *K* = 100.

No region exhibited a significant difference in HC versus BD using Family-Wise Error (FWE) with *p* < 0.05 in the *t* test in voxel by voxel analysis. However, when an uncorrected *p* value of 0.001 was used, two areas showed decreased GM ratios in the BD compared to the HC subjects. The relevant regions and MNI coordinates of the peak voxels are detailed in Figure 6 and Table 3.

**TABLE 3:** Gray matter alterations detected by voxel-based morphometry (VBM).

*P*-value	Contrast	Peak values location	MNI coordinates		
			*X* (mm)	*Y* (mm)	*Z* (mm)
*p* < 0.05 corrected	BD > HC	–	–	–	–
	BD < HC	–	–	–	–
*p* < 0.001 uncorrected	BD < HC	Left precentral gyrus	–48.48	10.25	35.48
		Middle frontal gyrus	27.80	33.36	36.75
	BD > HC	–	–	–	–

BD, bipolar disorder; HC, healthy controls; MNI, Montreal Neurological Institute.

## Discussion

To the best of our knowledge, this study is the first to explore alterations in striatal connectivity in patients with BD using static rsFC within a time window. The sliding windows were used to construct rsFC maps whose windowed striatal connectivity properties were assessed. We also explored the group differences in large time-scale connectivity, which was computed as correlations of fMRI time series over full-range of scanning time. In summary, large time-scale connectivity analyses did not reveal significant differences between BD patients and HCs. On the contrary, shorter time analyses presented BD patients with increased striatal connectivity in three time-windows (w69, w70, and w71). However, the variability of striatal connectivity did not demonstrate any significant difference between the groups. In Table 3, we compared the BD to the HC group using a two-tailed *t* test with a covariate of no interest (i.e., TIV). VBM analysis revealed no significant differences in GM volumes between BD and HC groups using *p* < 0.05 corrected. The opposite contrast produced the same outcome. While the BD group had a lower volume of GM in the Left precentral gyrus and Middle frontal gyrus than the HCs, no region was greater in the patients than the controls when adopting a *p* < 0.001 uncorrected and 100 extent threshold.

With respect to increased striatal functional connectivity observed in specific temporal windows, fairly similar findings to our observations were reported in a study of bipolar patients during reward processing, showing increased striatal connectivity to orbitofrontal cortex and amygdala (30). Lee et al. also found increased connectivity between the dorsal striatum and medial prefrontal cortex (mPFC) in bipolar patients with internet gaming disorder (69). Together, these findings suggest that patients with bipolar are characterized by altered striatal connectivity in large scale brain networks.

However, some previous studies could not identify increased connectivity associated with the striatum, but with other regions. One factor for this discrepancy could be that BD affects the brain differently in a selective population. For example, in the study by Syan et al., an obvious increase in functional connectivity was reported between the PCC and angular gyrus, and between the right dorsal lateral prefrontal cortex (dlPFC) and brainstem in women with BD attending clinical remission (70). Another selective study by Cerullo et al. showed an increase in the insula-to-right amygdala connectivity (71). In all these studies, the authors did not report any alterations suggestive of striatal connectivity attenuation.

Another factor may be attributable to the limitation of analysis methods. As changes in brain activity associated with disorders are not always vindicated in full-scale data. Rather, they are apparent in a short time-scale of seconds, and thus require short time-scale analyses to capture those patterns of deficits. In our study, increased striatal connectivity was observed in particular windows (w69, w70, and w71), but not across the entire time-series consistent with the aforementioned hypothesis. This is an important finding which was yet to be reported, suggesting that bipolar deficit-patterns can be evident in specific time-interval. Our results are in line with Nguyen et al. who demonstrated that shorter time scale provides more dimensions of brain functionality and dysfunctionality compared to full time-scale in BD (28, 72). In support of this, one study demonstrated that subtle changes in reoccurrence patterns of interactive intrinsic networks during cognitive tasks or at rest can be better modeled and detected in shorter time (42, 73). Together, these findings point to the conclusion that meaningful information that is likely to be lost in full time-scale can be trapped within a time window. To date, few studies have already been conducted in BD (28, 41, 43). Primary findings suggest aberrant insular (the right anterior) connectivity is related to abnormal salience processing (10, 74, 75), but more well designed studies are suggested to delineate brain neurological effects implicated by BD.

In this study, we detected a sole increase in striatal connectivity, but other studies have shown that BD can also lead to a decrease in striatal connectivity. Notably, the dorsal-striatum-to-middle-occipital-gyrus functional link in young males with internet gaming-related BD was weakened (69). Decreased functional connectivity in bipolar disorders was also reported linking other none striatal regions, specifically, the connectivity between the pregenual anterior cingulate cortex and amygdala, and between the ventral PFC and amygdala (76, 77).

Lack of patterns of reduced connectivity in our study could be connected to the type of BD. Here, all individuals were diagnosed with bipolar type I, which is likely to have lenient brain effects that allow the brain to reconfigure and strengthen its interaction as a compensatory mechanism than it could be in a severe level of bipolar such as type II. Specifically, the increased connectivity necessitated by BD can be thought of as the brain compensatory mechanism to recruit more resources to preserve, maintain, and restore cognitive functions or in response to cognitive demands which may have been detected during specific scanning time. In support of this hypothesis, a study of magnetic resonance spectroscopy identified higher concentrations of choline in the striatum in patients with BD than HCs (21, 78), suggesting the recruitment of more resources in this region.

However, in some studies, inconsistent findings have been reported in similar regions. Euthymic BD, for instance, showed greater connectivity between mPFC and right amygdala compared to HC (79), but these regions demonstrated reduced connectivity in another study (80). We hypothesize that individuals recruited in these studies had differential types (level) of bipolar leading to these inconsistencies, possibly reduced connectivity was related to a more severe level of brain injury associated with advanced BD. Methods to evaluate bipolar types should also be designed to be robust enough to delineate levels of bipolar effectively to reduce the chance of recruiting several types of BD patients in a single study. Other factors such as diverse samples and different preprocessing approaches could also lead to this inconsistency. Results show that the static FC in much smaller window significantly outperforms the full-scale analysis in terms of predictive accuracy. Static FC in smaller time series approach capture complimentary aspects of connectivity, and combining static and dynamic FC features will increase classification performance beyond what each type of feature can do on its own (43, 47, 48) and provides the local functional connectivity at each time window which is likely to capture important information that may be missed in either approach individually.

Fundamentally, BD is implicated in memory problems. The prefrontal cortex, which is involved in planning, reasoning, attention, problem-solving, and memory (81), is one of the targets, followed by the hippocampus associated with memory storage (82) and by the anterior cingulate cortex linked to emotional and cognitive functions (83). As blood flows into various brain regions the individual mood shifts (84). People may experience changes in update-and recall-working memory processes during and between episodes of BD (85). Studies have pointed out that difficulties in working and studying may also be experienced. Glahn et al. have shown executive function-related deficits that result in a reduced ability of planning and carrying out tasks of symptoms of BD (86), stress has demonstrated a strong relationship with changes in striatal activity. Coincidently, stress also correlated with learning performance, suggesting that it may alter the engagement of multiple memory systems (87) and the learning process. Of note is that the learning process involved the ventral striatum more in younger than older adults (88), which plays a key role in decision-making. Some findings from molecular and anatomical studies implicated the same regions in BD. Worth mentioning is the observed altered activity in the striatum during performing tasks involving balancing reward and risk. These studies, together, support that alterations found in striatal connectivity suggest bipolar-associated altered communication between the striatum and other brain regions. This conclusion supports the hypothesis that striatum is structurally, functionally, and chemically abnormal in BD.

We also document no difference in variability of striatal connectivity within each temporal windowed connectivity map between the two groups, this may be due to the normal aging effects on basal ganglia (89–91), suggesting substantial age-related abnormality that might interfere patterns of variability in the two groups. Our view is similar to that of Chakos et al. (19) who suggested that antipsychotics may alter blood oxygen level-dependent activations or anatomical structures in BP patients which could hinder the detection of the group differences. For example, a lack of a caudate volumetric difference between the HC and the BD group was linked with patients’ exposure to antipsychotics (92, 93). Although we did not achieve statistical significance in variability, it is possible that a better-designed study could provide more definite results.

When comparing our VBM results to those of other studies, in line with our findings, Seyedi et al. demonstrated GM reductions in the left precentral gyrus among other areas (67). Similar with our findings, Amyotrophic Lateral Sclerosis patients’ brains demonstrated less GM volume than those of controls on a voxel-level, at the right precentral gyrus and right middle frontal gyrus (94). However, it should be noted that the results of VBM in BD analyses are inconsistent. There were no significant variations in GM volumes between patients and HCs, according to several research (95, 96). While other research has found changes in the temporal and parietal gyrus (97) and frontal gyrus (98, 99), others found increased GM volume in bilateral precentral gyri in idiopathic blepharospasm patients compared to their respective matched healthy controls in another study (100). The reason for the inconsistency in BD or other illness may be because there are various subgroups, each with a different clinical manifestation but different causes and origins. Another reason also could be due to the fact that different techniques, statistical corrections, sample size, kernels, thresholds, and inclusion criteria were used. Generally, in BD, the observed GM volume anomalies are widely varied. The primary motor cortex, which controls voluntary movement, is anatomically located in the precentral gyrus (101). As demonstrated in BD patients, GM alterations in the precentral gyrus may impact primary motor cortex function, resulting in decreased control over voluntary movement. The dominant (left) middle frontal gyrus is involved in literacy development, while the nondominant (right) middle frontal gyrus is involved in numeracy development (102). To support this hypothesis, one study revealed that adolescents with remitted BD have a unique set of arithmetic problems that set them apart from both unipolar depression patients and HCs (103), the reason for this may be linked to neuroanatomical defects that cause cognitive impairments, such as a slower response time (103).

## Limitations

Our study, however, presents some limitations. First, initially we analyzed structural connectivity in the relevant regions that were identified in the functional connectivity analysis raising the possibility that structural abnormalities may be responsible for functional connectivity abnormalities in the disorders, unfortunately no significant differences were detected which led us to conduct whole-brain gray matter comparison, we believe that a better-designed study could provide more definite results. Second, the sample size was rather small which limits generalizability and thus, the larger sample size is required. Third, it is unclear whether the observed increase in striatal connectivity is specific to BD type I or shared by all types of BD. Fourth, the absence of clinical measures related to BD limits our results from evaluating the possible correlations between these patterns of deficits observed in bipolar and its clinical evaluations, hence attention should be paid when interpreting the results.

## Recommendations

According to the results of our functional neuroimaging analysis, understanding BD from the perspective provided by studies that look at the functional organization of the brain may significantly advance the neurological theory of this disorder. Over the past 10 years, tremendous progress has been made in our understanding of resting-state networks, both within and between individuals. While individual-level independent component analysis (ICA) or seed regions have frequently been utilized in techniques to identify resting-state networks, these analyses typically do not account for small but considerable variation among individuals in DMN and other networks. In fact, networks like the DMN may well be comprised of smaller sub-networks that underlie different cognitive functions (104). Despite these concerns, thorough group-level analysis application will probably result in better, more precise, and more systematic characterizations of network dysfunction not only in BD, but in other psychiatric disorders as well. The use of biomarkers in clinical practice is still underappreciated, and the data provided by biomarker research for clinical application is still unpersuasive, in contrast to the wealth of information available for medication research and development. Findings have been made for kynurenines (KYNs) and kynurenine pathway (KP) enzymes, which have been connected to a number of diseases including cancer, autoimmune diseases, inflammatory diseases, neurologic diseases, and psychiatric disorders (105–109).

## Conclusion

In this study, we looked into functional abnormalities involving the striatum between bipolar disorder patients and healthy controls and compare the morphological patterns of gray matter across the brain between the groups. Our findings suggest that bipolar illness is may be linked to striatal functional alterations and that, it is associated with a weakening of the precentral gyrus and middle frontal gyrus. Thus, striatal alterations, precentral gyrus and middle frontal gyrus are important in the pathophysiology of bipolar disease.

## Data availability statement

Publicly available datasets were analyzed in this study. This data can be found here: https://openneuro.org/datasets/ds000030/versions/1.0.0.

## Author contributions

CO, LQ, MJ, and HB made substantial contributions to the conception or design of the work, contributed to the acquisition, analysis, or interpretation of data, drafted the work, and revised it critically for important intellectual content. CO and LQ gave final approval of the version submitted. All authors contributed to the article and approved the submitted version.

## Footnotes

1
https://openneuro.org/datasets/ds000030/versions/1.0.0

2
http://rfmri.org/

3
https://www.fil.ion.ucl.ac.uk/spm/

4
http://www.nitrc.org/projects/gretna
